# Enhanced Feature Extraction and Integration with Advanced Machine Learning Algorithms for Sports-Injury CT-Image Classification

**DOI:** 10.2174/0115734056413312251111043047

**Published:** 2026-09-22

**Authors:** Wang Jun, Chu Huiqin, Rashid Abbasi, Md Belal Bin Heyat

**Affiliations:** 1 General Education Department, Anhui Xinhua University, Hefei 230088, China; 2 School of Mathematics-Physics and Finance, Anhui Polytechnic University, Wuhu 241000, China; 3 College of Computer Science and Artificial Intelligence, Wenzhou University, Wenzhou 325035, China; 4 School of Information and Communication Engineering, University of Electronics Science and Technology, Chengdu, China; 5 CenBRAIN Neurotech Center of Excellence, School of Engineering, Westlake University, Hangzhou, China

**Keywords:** Medical CT images, Image enhancement, Feature extraction, Machine learning, Medical imaging, Sports injury, RNNs, X-rays

## Abstract

**Introduction:**

X-rays, Computed Tomography (CT) scans, Magnetic Resonance Imaging (MRI), and ultrasound scans are examples of common medical imaging methods. These techniques are highly effective at extracting features, classifying, and diagnosing bone and muscle injuries. Enhancing athletic performance, minimizing injuries, and promoting players' overall health are the main goals of the vital discipline of sports medicine. Sports feature extraction of medical images, and sports medicine specialists help athletes reach their full potential while preserving their long-term health by preventing injuries and providing precise diagnoses.

**Method:**

We proposed a feature extraction approach for sport CT inquiry images, which were first preprocessed and then subjected to wavelet multi-scale analysis. The CT scan image of a sports injury is broken down into different directions based on smooth and rough regions. The Non-Down-Sampled Contour Transformation (NSCT) and Pyramid Bank (NSPFB) model is applied to the CT image of a sports injury. Furthermore, we employed Feedforward Neural Networks (FNNs), Convolutional Neural Networks (CNNs), and recurrent neural networks (RNNs) for medical image diagnosis. The model was tuned by using hyperparameters.

**Result:**

We proposed a feature-extraction model for medical CT scans of sports injuries and provided supporting statistics for medical diagnosis. The CT scan image of the sports injury is subdivided into different sub-directions based on smooth and rough regions. Furthermore, enhanced sub-regain is achieved using an adaptive gamma correction and multi-directional image enhancement *via* wavelet layering. Our proposed method has demonstrated a remarkable accuracy of 88%, while the deep learning model TabNet-Transform has achieved an imposing 90%. The proposed algorithm improves the quality of the output images and is computationally efficient.

**Discussion:**

Current research indicates that the most vital area of medicine is image analysis, which helps radiologists to make more accurate diagnoses. The feature extraction process involves extracting valuable information from higher-level representations, such as the Region of Interest (ROI) for image analysis, and transforming the image from these representations into lower-level pixel data. The performance analysis of the proposed scheme yielded an efficient extraction time. When extracting feature characteristics for 10 to 30 medical CT images of sports injuries, the average computation time is 2ms to 6 ms.

**Conclusion:**

This research explored the application of machine learning and advanced artificial intelligence models to predict disease diagnosis and to enhance feature extraction from medical CT images of sports injuries. However, several variables impact the original image, including removing image noise and detecting specific regions or objects of interest. Our findings pave the way for further investigations of sports injury and early disease prognosis. We may incorporate data from wearable devices to improve their predictive power and extract the desired features for disease prognosis analysis.

## 1 INTRODUCTION

X-rays, Computed Tomography (CT) scans, Magnetic Resonance Imaging (MRI), and ultrasound scans are examples of common medical imaging methods. These techniques are effective for feature extraction and for classifying and diagnosing bone and muscle injuries. Enhancing athletic performance, minimizing injuries, and promoting players' overall health are the main goals of the vital discipline of sports medicine [1-3]. Sports feature extraction of medical images, combined with the expertise of sports medicine specialists, helps athletes reach their full potential. Preserving athletes’ long-term health involves preventing injuries, providing precise diagnoses, and providing individualized restoration plans.

Sport feature extraction will remain at the forefront of encouraging safe and sustainable athletic participation as the discipline advances with new knowledge and technological breakthroughs. To minimize the incidence and severity of inaccurate diagnoses, it is essential to identify individuals at high risk of developing these diseases and initiate appropriate treatments. Nowadays, there has been increasing interest in Artificial Intelligence (AI) and Machine Learning (ML) to predict and accurately diagnose diseases, and to analyze them using advanced tools [4]. They enable customized risk evaluations and therapies for every patient by combining several risk factors. Medical personnel can employ AI-powered prediction models to improve decision-making and resource allocation. These tools can yield better patient care and overall community health outcomes. Large, complicated data sets analyzed by AI tools can also reveal patterns that are not easily detectable by humans, making them more effective. We aim to provide long-lasting, personalized therapies by extracting real-time data and improving predictive models. Currently, block-based segmentation, edge matching, and grayscale detection are the primary feature extraction techniques in sports injury analysis. To achieve detailed feature extraction of sports injury actions, the image's active contour is identified, and the contour and edge feature points are precisely calibrated and segmented; however, complications arise due to inadequate anti-interference dimensions and low convergence [5, 6]. A graph Convolutional Neural Network (CNN) based technique for identifying actions for aerobics sports injuries was proposed in the literature [7-9]. It splits the aerobics video into multiple images, removes the background from the aerobics sports injury action image, and establishes a grayscale co-occurrence matrix that estimates the local-area fuzzy kernel and visual error of the image. Furthermore, the author achieves feature extraction and action recognition accuracy. The directed skeleton time graph representation and time-series information modelling of the human body are trained and implemented using the CNN model [10-12]. However, this scheme has low extraction efficiency because it takes a long time to extract image data. The complexity of sports injury image analysis, which involves challenging image processing tasks such as feature identification, tracking, and recognition, is the main cause of this problem. Deependra *et al*. [13] proposed a DL-based tumour-feature extraction model for ROI-based tumour segmentation, inspired by a 3D neural network. Several variables, such as tobacco and alcohol use, allergies, physical activity, infections, viral infections, and climate change, contribute to the severe progressive disorder known as skin cancer. Puneet *et al*. [14] proposed a hybrid U-Net model using Machine learning to segment and classify the skin cancer disease [15, 16]. The hybrid approach enhances the performance and efficiency of FrCN for U-Net segmentation and feature extraction. The R-CNN algorithm further improves model performance and classification accuracy, and enhances the differentiation between malignant and benign tumors [17]. These techniques can be laborious and computationally demanding, as they often use complex algorithms and lengthy calculations to examine feature-extraction sequences frame by frame. The main purpose of this study is to determine how well machine learning models perform in extracting, predicting, and enhancing CT image features and other sensor data to improve the diagnosis of complex diseases. The contribution of this study is as follows: To develop an ML-based feature extraction model to provide personalized, early diagnosis and reduce the risk of sports injuries. Developed an enhanced CT image feature extraction that encompassed demographics, medical history, lifestyle, and environmental factors.

- Compared to ML models, RF and Tab-Net extract their features with high efficiency and precision.

## 2 MATERIALS AND METHODS

Current research specifies that the most vital area of medicine is image analysis, which helps radiologists to make more accurate diagnoses. Feature extraction, extracting valuable information from higher-level representations, Region of Interest (ROI) for image analysis, and transforming higher-level representations into lower-level pixel data. Li *et al*. [18] proposed an automatic method for extracting features from CT images. The distribution of directed gradients in a chosen area is reflected in the statistical feature descriptor based on a directed-gradients histogram. Furthermore, the author used an AdaBoost classifier, which outperformed the hybrid technique (76%) and the edge-based method (45%), achieving an accuracy of 84%. The study employed the Scale-Invariant Feature Transform (SIFT) technique to extract local features from the ROI and eliminate the remaining background region [19]. The Author follows a background denoising scheme, key-point extraction using SIFT, ROI detection using SVM, and a sliding window. The author achieved 96.57% accuracy with a neural network classifier based on the SIFT algorithm, which is 3.44% higher than the SVM's classification accuracy. One promising technique uses level Co-occurrence matrices to extract texture features based on the distribution of pixel intensities [20]. Level Co-occurrence Matrix characteristics and feature extraction are as follows. Feature extraction: contrast measures the variation in intensity difference between a pixel and its neighbors, calculated using Eq. (**1**).


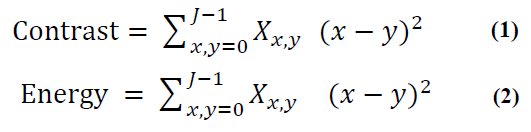


The Gray-Level Co-occurrence Matrix (GLCM) is used for texture analysis, and the matrix values fall between 0 and 1, calculated using Eq. (**2**), while the grayscale levels are distributed uniformly throughout the image, and the grey-level correlation of neighbouring pixels is determined using Eqs. (**3** and **4**):


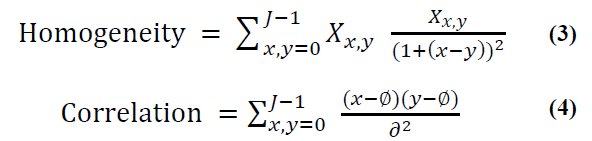


The cosine transform is used to alter images in Discrete Cosine Transform (DCT)-based feature extraction [21] and assigns high-energy coefficients to low-energy areas. After applying the cosine transform to a 2D image, most of the energy is stored in a comparatively small number of DCT coefficients. Furthermore, the lower-order DCT coefficients use a larger bit count, while the higher-order coefficients are properly reduced using fewer bits. Feature values of the cosine transformation are computed using Eqs. (**5** and **6**).


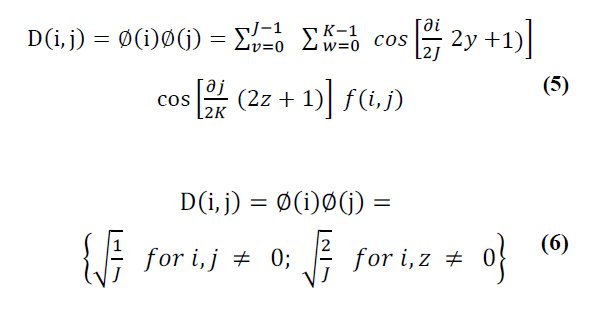


Consequently, y and z are pixel intensities of the image.

The Haar transform uses low-pass and high-pass filters in a wavelet transform, splitting the input image into various frequency bands. 2-D transform returns the detailed estimated coefficient matrices [22]. The mean, entropy, and standard deviation are computed using these coefficients, and the results are considered feature vectors that are modified using the Discrete Wavelet Transform (DWT) defined by Eqs. (**7** and **8**).


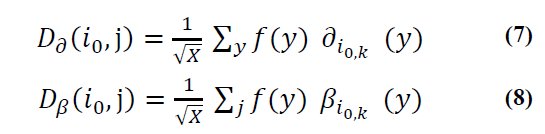


Where ƒ(*y*) ∂*_io_*_,_*_k_* (x) and *β_io_*_,_*_k_* are functions of variables y.

Sports injuries in medical CT scans are a critical tool for visualizing information that provides graphical data to the doctor for diagnosis and treatment, especially for radiologists and physicians [23, 24]. However, several variables affect the original image and require removing image noise and detecting regions or objects of interest, as shown in Fig. (**1**). A series of band-pass multi-resolution filters is the counterpart of wavelet decomposition and breaks down CT scans of sports injuries into smaller sub-images. The set *α*(*w*) and *b*(*w*) filters are used to extract features from medical CT images, and to subdivide them into four sub-band images *H*_1_, *H*_2_, *H*_3_ and *H*_4_ which are then extracted from the original images.

The sets of *α*(*w*) and *b*(*w*) filters are grouped together; the original image is subdivided into 4 sub-bands: *H*_1_, *H*_2_, *H*_3_ and *H*_4._ For example, let *α* be the wavelet function, and *β* the corresponding scaling function; the wavelet decomposition is expressed as Eqs. (**9**-**11**).


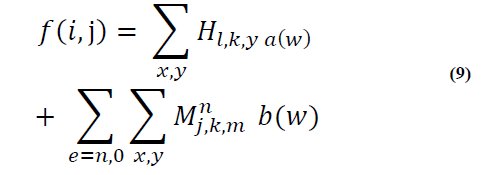



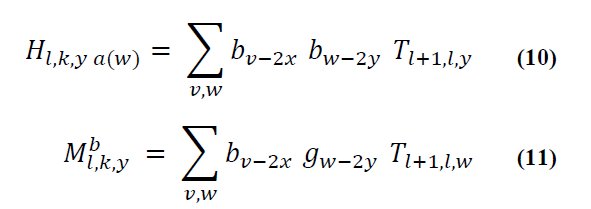


The low-frequency coefficient for CT imaging of sports injuries is represented by the M*_l_^b^,_k,y_*. The sports injuries in the horizontal, vertical, and diagonal directions are denoted as M*_j_^n^,_k,m_* while *b_v-2x_* and *g_w_*_-2_*_y_* represent the low-pass filter's coefficients.

### 2.1 Image Enhancement

Due to low or dark illumination, vital diagnostic information cannot be detected in sports injury images. Therefore, to maintain colour information in the improved images, the luminosity enhancement process is properly performed by calculating the luminance values using the gain matrix *E_N_*(*µ*, *π*), as shown in Eq. (**12**).


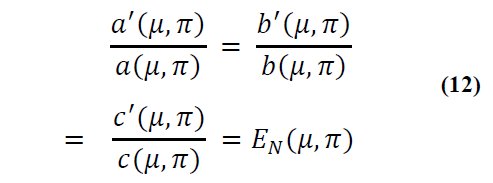


The R, G, and B color channel values of a pixel location (*µ*, *π*) in the original and enhanced image. The intensity matrix for colour enhancement is given by Eq. (**13**).


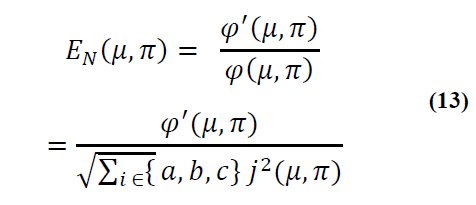


Where 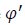
(*µ*, *π*) is the contract enhancement and intensity of the image at the pixel position *ϕ*’ (*µ*, *π*) while increasing the contract used, Adaptive Gamma Correction (AGC) of input images [25]. This is a computationally efficient technique for increasing the perceptibility of all kinds of images. The cumulative distribution function of the normalized histogram of the input image is used to determine the gamma correction weight. Image enhancement can also be performed in different directions for CT images of sports injuries after wavelet decomposition, with the gray-level values in a flat region adjusted, thereby enhancing image quality and expanding the low-frequency portion [23]. To better retain the fine details of CT images, edge-denoising enhancement can be applied to images in various orientations using wavelet sparseness.

### 2.2 NSCT-GLCM Based Feature Extraction

In selecting appropriate structural elements for the mathematical morphology edge detection operator, it can be stated that clear-cut image edges are obtained by applying basic morphological operators, such as dilation, erosion, morphological opening, and morphological closing [26]. For edge extraction in CT images, suitable structural elements are selected that are commensurate with the image's texture. Furthermore, it is also essential to consider the size, shape, and orientation of the structural elements. Usually, 3 × 3 or 5 × 5 square structural elements are selected, while others are chosen based on the specific experience and requirements. The detailed steps of the NSCT-GLCM pipeline are shown in Fig. (**2**). As per the final results about the relationship of a morphological operation, corrosion, and dilation, it is justified within the medical CT images made for a sport tear injury as follows: As per the final results about the relationship of a morphological operation, corrosion, and dilation, it is justified within the medical CT images made for a sport scratch injury as follows (Eq. **14**):


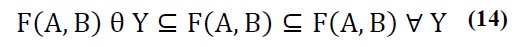


Wherein θ and 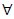
are such arithmetic symbols, in which F(A,B) gives the enhanced grayscale medical CT image of a sports tear lesion. Y denotes the gray structural element for this enhanced medical CT image of a sports injury. Morphological operations opening and closing are determined as in Eq. (**15**):


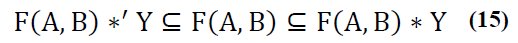


F(A,B) _*_’Y denotes an operation in morphology, whereas F(A,B) _*_’Y denotes a closed operation in morphology. The process of edge extraction in CT images based on extension mathematical morphology for sport-related scratch injuries can be described as follows: Let Di(F) denote a dilation residue edge detector. It is defined as: the difference image between the genuine medical CT image F(A,B) and expansion by structural element Y: (Eq. **16**)


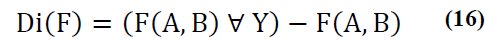


In the same way, let Di(F) be a residual edge detector. It is defined to represent the difference, in terms of the original image and image F(A,B), which is etched by structural element Y, as defined in Eq. (**17**):


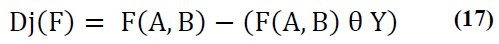


The morphological gradient D(F) of the image F(A,B) is obtained through dilation and erosion operations. (Eq. **18**):


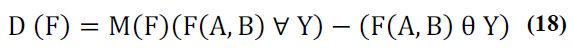


Here, F(A,B) 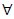
Y indicates an open operation. Y is a structural element of 3 × 3. Morphological opening and closing operations, used to filter noise, were applied as preprocessing. Then, the morphological opening operation is performed to smooth the image. At last, the expansion operation is done. The difference between the expanded and unexpanded images results in a sharper edge. M(F) is the resultant edge-extraction image for medical CT images of sports injuries.

### 2.3 Non-downsampled Contour Transformation

NSCT comprises two components: a Pyramid Filter Bank (NSPFB) and Directional Filter Banks (NSDFB). The NSCT segmentation can be elaborated. First, the NSCT operates on the extracted edge M(F) for multi-scale segmentation of medical CT images of sports injury. High-frequency subband images, as well as a low-frequency subband image, are derived at various frequencies.

The NSDFB is then applied to the high-frequency sub-band images using multi-directional transformations. This allows the different sets of sub-band images to be acquired at different scales and orientations [27, 28]. Linear distance between two-pixel locations where the gray value for CT images of sport injury for a different pixel whose gray value at an angle of θ is A. The probability of D(A, B, d, θ), co-occurring in the entire medical CT image of sports tear injury, is defined in Eq. (**19**):


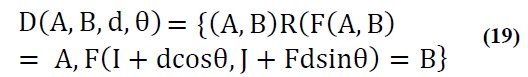


The variables *I* and *J* refer to medical CT image parameters for sports injuries. The classic texture features for medical CT images of sports injuries are the homogeneity measures: Angular Moment (AM), Entropy (ET), and Dissimilarity (DS). The terms are defined in the following equations. (Eqs. **20**-**23**).


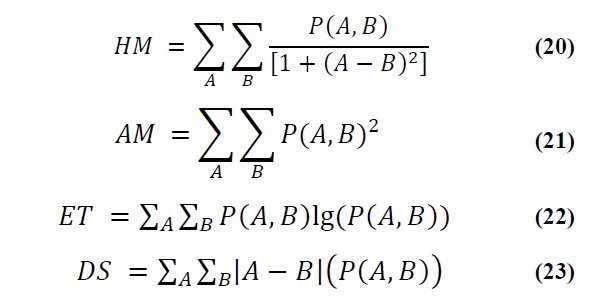


### 2.4 NSCT Segmentation for Medical CT Images of Sports Injuries

The edge-extraction M(F) is used to subdivide an image into two layers *via* the NSCT. A medical CT image is then decomposed into sub-bands with different scales and directions [27, 29]. These are 13 sub-bands, all with the same dimensions as the original image and representing a field. A sub-band represents each texture in a given direction. The NSCT low-frequency sub-band Sb would approximate it to a uniform distribution. The high-frequency sub-band gives a distribution of high peaks and long tails. Low-frequency sub-bands of medical CT images of sports tear injuries are uniformly quantized. This quantization is a 16-analyzed quantization level according to Eq. (**24**), as follows:


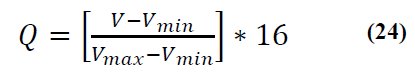


While Q denotes quantization, V is a quantization vector, *V_min_* is the minimum value of a quantization vector, and *V_max_* is the maximum value for a quantization vector. In this case, the medical injury image detail sub-band is used. Quantization of coefficients occurs at 16 levels. Initially, estimate the variance *δ_v_*. Each sub-band at the same scale is subjected to +3*δ* segmentation quantification using Eq. (**25**).


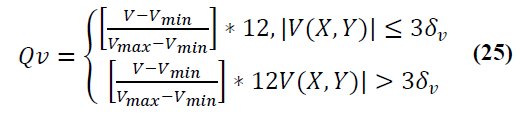


There will be different numbers of segmentation directions due to the variation in the NSCT segmentation constraints. The difference in the scales will differ with the variation in the direction limit as well. The symbiotic sum is extracted from the GLCM using Eqs. (**19**-**22**). Therefore, four symbiotic quantities of HM, AM, ET, and DS are calculated separately for each sub-band of NSCT at angles 0, 45, 90, and 135 degrees.

## 3 RESULTS

To validate the efficiency of the proposed algorithm in this paper, 1200 CT images were chosen for training and feature selection in the Image Reconstruction for Comparison of Algorithm Database(3D-IRCADb-01)database (https://www.ircad.fr/research/3d-ircadb-01/, https://pmc.ncbi.nlm.nih.gov/). This paper tests and evaluates the algorithm's efficiency. The experimental platform uses an NVIDIA GeForce RTX 3060 GPU, an i7, and 16 GB of RAM. The experiments' data come from various sources, including open-source databases. The image enhancement and noise removal using several steps are defined in the previous section. The training set is 60%, the 20% validation set is used for hyperparameter tuning, and the remaining 20% is used for the test set. The original CT image number of variables during the acquisition of sports injury image data deteriorates, which demands image enhancement processing algorithms. Poor-quality sports injury images make it difficult to diagnose the condition. The proposed algorithm improves the quality of the output images, as shown in Fig. (**3**). The diagnostic effectiveness of a situation in image analysis depends on the effectiveness of CT image analysis and the value of other check-up reports. The 100 individuals out of the 30 individuals with sports injury images and their other circumstances were evaluated, and the extracted features (smooth and rough regions) of medical CT imaging [21, 30] were used. The proposed scheme achieved efficient extraction time when extracting feature characteristics from 10 to 30 medical CT images of sports injuries; the average computation time was 2-6ms, as shown in Fig. (**2**). The author [31] reported extracting image features in 21ms and 30ms. A previous study [32] reported extraction times of 45ms for image features and 2ms for 7 CT images of sports injuries [27]. In image pre-processing, we use imputation or removal to handle missing values. We also used label encoding to translate categorical data (such as gender and ethnicity) into numerical values. Scaling numerical features to a constant range ensures that the model's predictions are not greatly affected by a single feature. We used Feed-Forward Neural Networks (FNNs), Convolutional Neural Networks (CNNs), and Recurrent Neural Networks (RNNs) for medical image diagnosis. We have tuned our model using hyperparameters. Hyperparameter tuning is the process of adjusting a model's parameters to improve performance [33, 34].

## 4 DISCUSSION

This study detailed a preprocessing algorithm for carefully selecting a set of model constraints and demonstrated the outcomes of feature extraction, image segmentation, and classification tests. Preprocessing steps were involved to improve the precision of CT and MR images. The main objective is to convert CT images into another format that can be adequately processed by a machine learning system or a doctor for further diagnosis. Preprocessing steps not only enhance the signal-to-noise ratio but also improve the visual quality of CT sport injury images, eliminate annoying background elements and noise, and smooth the interior and edge regions. Training, testing, and validation (60%, 20%, and 20%) of CT sport injury images for the experiment due to limited memory and datasets for training, testing, and hyperparameter tuning. Similarly, GLCMs with different window sizes are adopted for different NSCT segmentation scales [35]. For medical CT images of sports injuries, the sub-band image scales for the first and second sub-bands are *Y* = 3×3 and 5×5, respectively. (Eq. **26**)


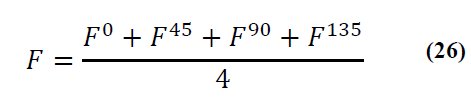


Angle orientation and distance produce pixel information from the GLCM scheme that classifies the texture of an image. The pixel information is too close to the sensor, which means each piece of information tends to be similar, and the pixels are inappropriate; the information between adjacent pixels extends too far. For example, we defined the distance value and generated settings for four angles: 0, 45, 90, and 135 degrees. Several variables of information are extracted using the GLCM feature extraction process. The GLCM used dissimilarity, contrast, correlation, and energy factors to analyze an image's texture.

Predictive modelling and classification challenges were addressed using the K-Nearest Neighbors (KNN) supervised machine learning method. KNN assigns the test data to the class label with the smallest distance to each training point [36]. The maximum N points that are similar to the test data are chosen, while the training data class with the highest possibility is chosen for the test data using the KNN algorithm. Support Vector Machine (SVM) has lower accuracy (0.73) than K-Nearest Neighbors (KNN) (0.80), signifying they may not be suitable for this dataset. Random Forest performs better than other models, achieving accuracies of 0.89, 0.88, and 0.93, and precisions and recalls of 0.88 and 0.93, respectively, as shown in Table **1**.

Convolutional Neural Networks (CNN) perform better because they’re able to get spatial pattern associations in images, therefore achieving high accuracy of the CNN model (92.40) in the image dataset, while the accuracy, precision, and sensitivity are 0.84, 0.83, and 0.81, respectively, due to the different nature of the data, revealed in Fig. (**4a** and **b**). Models perform better and can process data in order while finding patterns in time series, for example, patient history [37]. Tab-Net-Transformer performed better than all other models, with an accuracy gain of 0.90. It is well-suited to highly structured data, but underperforms on image or unstructured data. RNN models are less appropriate for image data; however, they perform better at extracting sequential dependencies within slices. The GAN and DQN achieve 0.77 and 0.85 accuracies, which are slightly lower than those of another model shown in Table **2**. Choosing the optimal DL model is challenging for achieving optimal results in the healthcare field. Carefully select a model that meets specific requirements and can handle diverse data types. The Random Forest and TabNet models estimate ROC curve scores. Tab-Net performs better in AUC scores than Random Forest, which efficiently discriminates between false positives and false negatives. In the Precision-Recall (PR) curve analysis, the Tab-Net model also achieves better precision and recall than the DQN, RNN, and GAN algorithms. Tab-Net model effectively reduces both false negatives and false positives and performs better on structured data, as shown in Fig. (**5a**-**d**). Tab-Net effectively minimizes false positives and false negatives, as well as the ROC and PR curves, which are important for precise diagnosis.


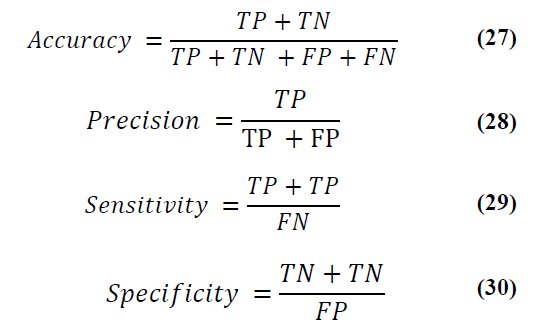


The accuracy metric is the total prediction by the algorithm, as expressed in Eq. (**27**); precision, sensitivity, and specificity are expressed in Eqs. (**28**-**30**), respectively. The statistical technique used to assess the model's effectiveness across all classes is known as cross-validation, which ML uses to evaluate its algorithms. Each class is given an equal weight, and before validating, which comprises all samples of the data and trains the algorithm, the data is partitioned into two parts. The K-fold method: repeat the training and validation process K times, with each fold used for validation in turn.

CNN models have outperformed in numerous classification problems in the past, but CNN architectures remain challenging because they perform differently across the same dataset. The author [17] employed the CNN model on the Alzheimer's disease dataset. The dataset contains 6400 images: 3200 with dementia and 3200 without dementia. Deependra proposed [13] a DL-based unified MRI brain tumor examination, feature extraction, and segmentation. The author precisely segments brain tumors from unfired MRI scans and predicts glioma survival in patients. For training, testing, and validation, the author partitions into three portions (75%, 13%, and 12%), respectively. Arfat *et al*. [15] proposed a CNN model to detect Omicron-caused pneumonia in radiology images (COVID-19 infection types). COVID-19 has variants that target different patterns of pneumonia. The author's proposed model successfully classifies COVID-19-caused pneumonia, normal pneumonia, and Omicron pneumonia from radiology images. The ratios of accuracy, specificity, and sensitivity were 78, 79.81, and 78.45. The author proposed [38] ML classification of CT images and GLCM-based feature extraction for COVID-19 screening. The author extracts GLCM features from a chest CT image using an ML algorithm. K-nearest neighbors, SVM, and the Random Forest classifier are used to collect the pattern, and hyperparameters are normalized by tuning tests. Random Forest and SVM achieve the best results, reaching up to 99.94% accuracy on GLCM features. The proposed method for CT Scan sport injury feature extraction based on NSCT-GLCM using ML models (Random Forest and Tab-Net-Transformer) performed better than all other ML models, achieving an accuracy gain of 0.89-0.90. It is well-suited for highly structured data, but underperforms on unstructured data.

## 5 STUDY LIMITATIONS

There are limitations to the use of quantitative training load indicators for predicting injury risk. The volume of data that training analysis and extraction can provide for injury risk prediction is limited, since injury risk is caused by multiple factors. Furthermore, there is a challenge with a large amount of mishandled data and the lack of a consistent validation technique.

## CONCLUSION

This research explored machine learning models to predict disease diagnosis and to enhance feature extraction from medical CT scans of sports injuries. We proposed a feature-extraction approach for sport CT injury images, first preprocessed and then subjected to wavelet multi-scale analysis. The CT scan sports injury image is decomposed into different directions based on smooth, high-frequency regions and rough, low-frequency regions. To achieve multi-scale improvement for CT sport injury images, multi-directional frequency-region enhancement and an adaptive gamma-correlation are applied using wavelet layering to expand dark or low-illumination regions. Our findings pave the way for further investigations of sports injury and early disease prognosis. In the future, we may include data from wearable devices to improve their ability to forecast risk and extract the desired features for disease prognosis analysis. Future work should emphasize enhanced interoperability across various clinical organizations and the optimization of algorithms.

## Figures and Tables

**Fig. (1):**
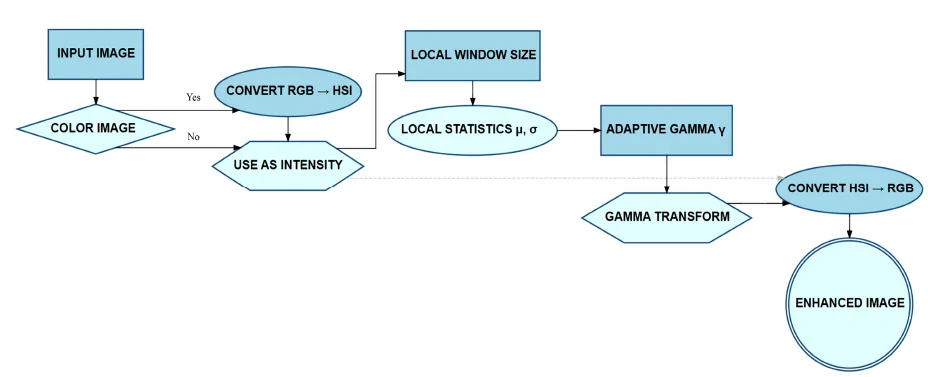
Flowchart of the preprocessing pipeline of image enhancement and edge denoising. The process of contract enhancement and image intensity at the pixel level, increasing contrast using adaptive gamma correction of input images.

**Fig. (2):**
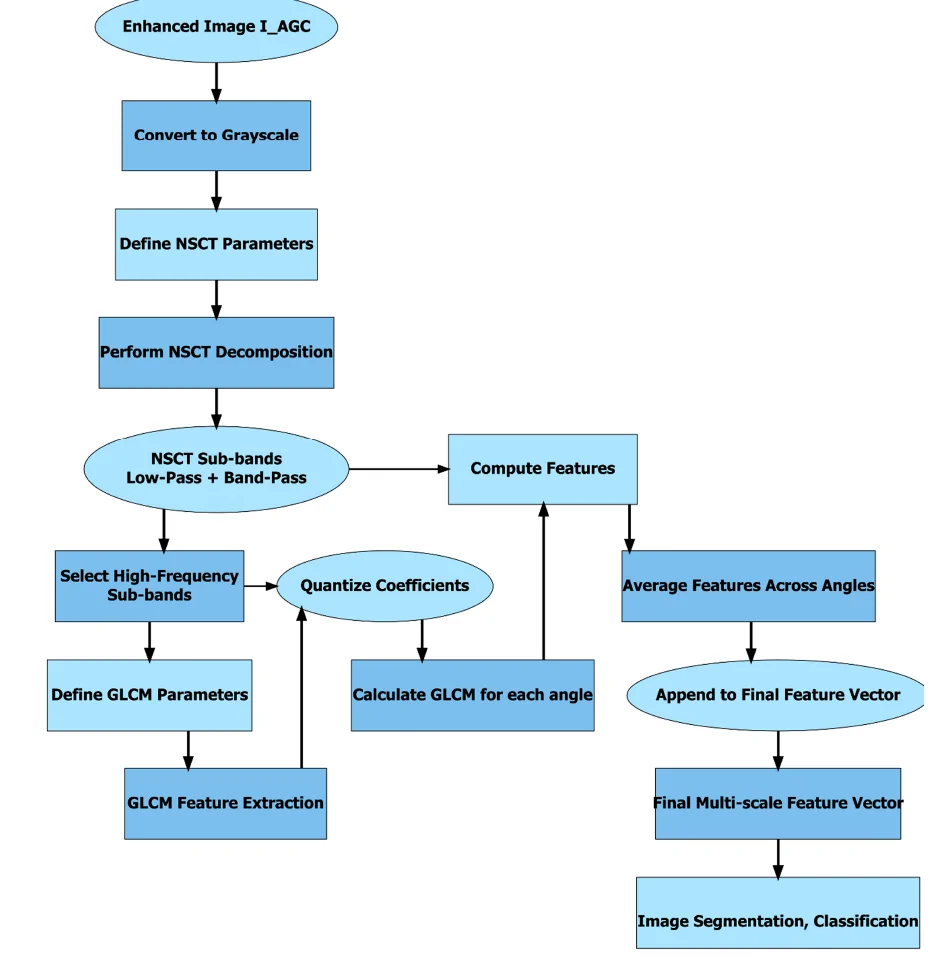
Block diagram of the multi-scale and multi-directional image decomposition process. Non-subsampled contourlet transform and gray level co-occurrence matrix for feature extraction.

**Fig. (3):**
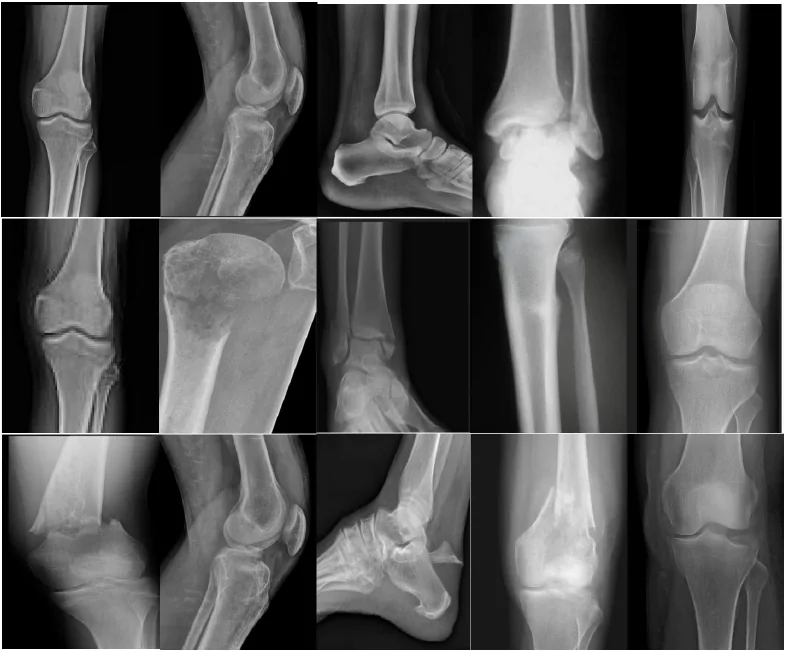
Enhanced images of sports injury images.

**Fig. (4):**
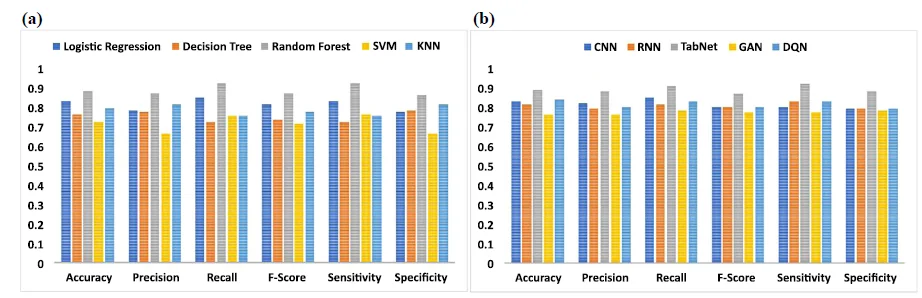
(**a**) Predicted accuracy comparison of the following machine learning and deep learning models: decision tree, random forest, SVM, KNN, CNN, Tab-Net, and GAN. (**b**) Performance metrics of the following deep learning models (CNN, RNN, Tab-Net, DQN, GAN) for precision, recall, and F1 score.

**Fig. (5):**
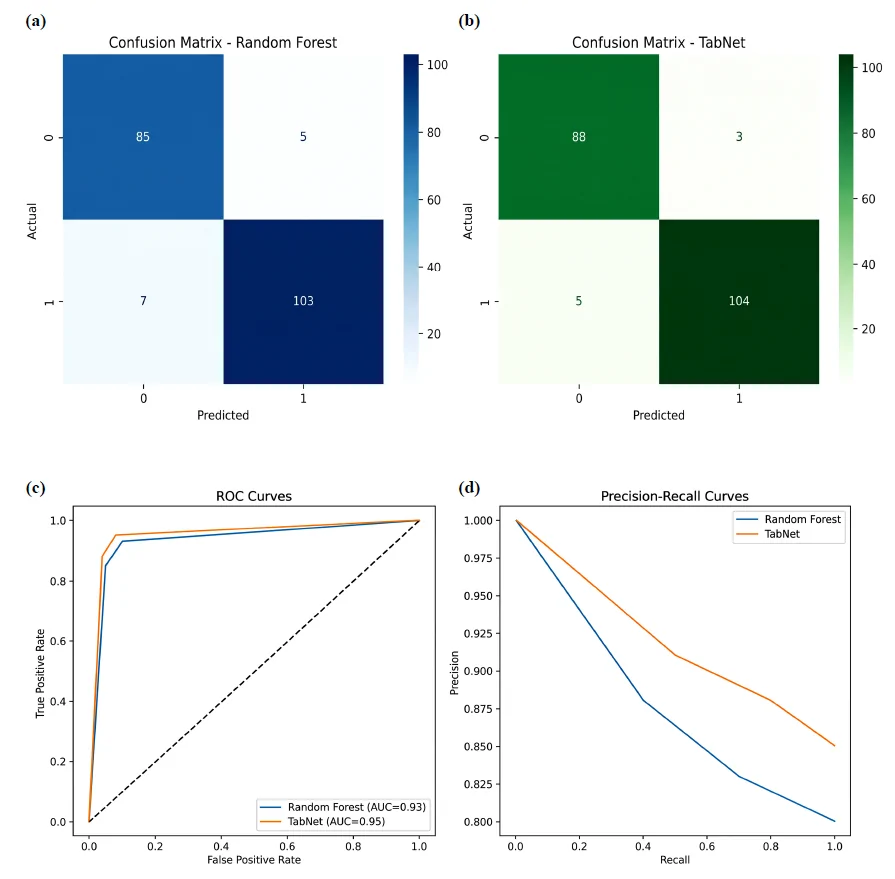
Predicted accuracy comparison of the following machine learning and deep learning models. (**a**) confusion matrix for random forest (**b**), confusion matrix for Tab-Net (**c**), and ROC curve for the random forest and Tab-Net models. (**d**) Precision recall curves for random forest and Tab-Net Mode.

**Table 1:** Performance matrix (accuracy, precision, sensitivity, specificity, *etc*.) of machine learning models.

ML Models	Accuracy	Precision	Recall	F-Score	Sensitivity	Specificity
Logistic Regression	0.84	0.79	0.86	0.82	0.84	0.78
Decision Tree	0.77	0.78	0.73	0.74	0.73	0.79
Random Forest	0.89	0.88	0.93	0.88	0.93	0.87
Support Vector Machine	0.73	0.67	0.76	0.72	0.77	0.67
K-Nearest Neighbors	0.80	0.82	0.76	0.78	0.76	0.82

**Table 2:** Performance matrix (accuracy, precision, sensitivity, specificity, *etc*.) of Deep learning models.

Deep Learning Models	Accuracy	Precision	Recall	F-Score	Sensitivity	Specificity
Convolutional Neural Network (CNN)	0.84	0.83	0.86	0.81	0.81	0.80
Recurrent Neural Network (RNN)	0.82	0.80	0.82	0.81	0.84	0.80
TabNet- Transform	0.90	0.89	0.92	0.88	0.93	0.89
Generative Adversarial Network (GAN)	0.77	0.77	0.79	0.78	0.78	0.79
Deep Q-Network (DQN)	0.85	0.81	0.84	0.81	0.84	0.80

## Data Availability

All data generated or analyzed during this study are included in this published article.

## LIST OF ABBREVIATIONS

MRI: Magnetic Resonance Imaging
CT: Computed Tomography
NSCT: Non-Down-Sampled Contour Transformation
AI: Artificial Intelligence
CNN: Convolutional Neural Network
